# Hepatoprotective effects of astaxanthin and kokum on lipid peroxidation and fatty acid profile in mice with diet-induced NAFLD

**DOI:** 10.3389/fphar.2026.1841100

**Published:** 2026-06-19

**Authors:** Natalia Ksepka, Kamil Wysocki, Maima Matin, Karolina Lach, Atanas G. Atanasov, Jarosław O. Horbańczuk, Artur Jóźwik

**Affiliations:** 1 Department of Biotechnology and Nutrigenomics, Institute of Genetics and Animal Biotechnology of the Polish Academy of Sciences, Jastrzębiec, Poland; 2 Division of Medical Biology, Institute of Biology, University of Jan Kochanowski, Kielce, Poland; 3 Ludwig Boltzmann Institute Digital Health and Patient Safety, Medical University of Vienna, Vienna, Austria; 4 Department of Biochemistry, Saveetha Medical College and Hospital, Saveetha Institute of Medical and Technical Sciences, Chennai, Tamil Nadu, India; 5 Patient Safety & Digital Health (PaDiH) Group, Danube Private University, Krems-Stein, Austria

**Keywords:** astaxanthin, fatty acids, kokum, lipid peroxidation, MASLD, NAFLD

## Abstract

**Introduction:**

Among adults, there is a constant increase in the incidence of non-alcoholic fatty liver disease (NAFLD). The significant limitations of available pharmacological therapies for this condition have led to intensive research into natural bioactive compounds with hepatoprotective properties. Therefore, this study focused on evaluating the effects of astaxanthin and kokum supplementation on lipid peroxidation levels, fatty acid profile, and lysosomal activity in mice liver with diet-induced NAFLD.

**Methods:**

The study was conducted using 120 male Swiss Webster mice. A methionine- and choline-deficient (MCD) diet was applied to induce hepatic steatosis. Following steatosis induction, animals received astaxanthin, kokum, or combined supplementation for 90 days. Hepatic fatty acid profile, malondialdehyde (MDA) levels, and lysosomal enzyme activities were subsequently evaluated. Statistical analysis was conducted using one-way ANOVA followed by Tukey’s *post hoc* test.

**Results:**

Hepatic steatosis was associated with the accumulation of saturated fatty acids (SFA), elevated MDA concentration, and increased lysosomal esterase (EL) and lysosomal acid lipase (LAL) activities compared with the control group. The proportion of SFA increased from 37.1% in controls to 45.8% in steatotic animals. Supplementation with astaxanthin and kokum promoted a higher degree of fatty acid unsaturation and reduced lipid peroxidation. The DAK group, representing steatotic mice receiving combined supplementation, showed the lowest proportional SFA content among all experimental groups (29.7%) and the lowest MDA concentration among steatotic groups. In addition, supplementation contributed to partial normalization of lysosomal enzyme activities.

**Conclusion:**

Supplementation with astaxanthin and kokum, especially in combination, demonstrated a synergistic protective effect by increasing the degree of fatty acid unsaturation, reducing lipid peroxidation, and normalizing lysosomal activity. These findings indicate the therapeutic potential of multi-targeted nutraceutical strategies in the prevention and treatment of NAFLD.

## 1 Introduction

The liver is an important metabolic organ, and its energy metabolism is tightly regulated by a wide range of nutritional, hormonal and neuronal signals that coordinate glucose, lipid and amino acid homeostasis. In addition, to using glucose as a metabolic fuel, the liver also converts glucose into fatty acids (FA) and serves as the main reservoir of lipid fractions (Rui, 2014). Under physiological conditions, hepatocytes effectively process free fatty acids (FFA), synthesize triglycerides (TG) and cholesterol esters (CE), and then secrete them into the circulation in the form of very low-density lipoproteins (VLDL) (Heeren and Scheja, 2021).

Disruption of lipoprotein metabolism in the liver leads to excessive lipid accumulation in hepatocytes and contributes to systemic consequences. One of the most common metabolic diseases, associated with lipid fractions in the liver, is non-alcoholic fatty liver disease (NAFLD), a condition increasingly referred to as metabolic dysfunction-associated steatotic liver disease (MASLD) (Huang et al., 2025). Due to the ongoing transition in nomenclature and the fact that the present study was initiated before the implementation of the updated diagnostic criteria and terminology, the term NAFLD has been retained throughout this manuscript. Furthermore, previous evidence indicates that findings obtained under the former nomenclature remain valid and applicable in the context of MASLD (Israelsen et al., 2024; Yagüe-Caballero et al., 2024; Younossi et al., 2024).

NAFLD affects more than 25% of the adult population. Its characteristic feature is the excessive accumulation of triglycerides in hepatocytes, which leads to inflammation and fibrosis. Untreated NAFLD can progress to liver cirrhosis, and in advanced stages, to hepatocellular carcinoma (Buzzetti et al., 2016; Svobodová et al., 2025). Oxidative stress and lipid peroxidation are key factors in disease progression, resulting in the formation of toxic aldehydes such as malondialdehyde (MDA), which contribute to damage of cellular membranes, organelles and DNA (Arya et al., 2021; Wang and Liu, 2025).

Lipid peroxidation products not only serve as biomarkers of oxidative damage, but also act as active signaling mediators that promote inflammatory reactions and apoptosis, affecting the transition from NAFLD to non-alcoholic steatohepatitis (NASH) (Arroyave-Ospina et al., 2021; Martín-Fernández et al., 2022). Lipotoxicity associated with excess FFA further impairs lysosomal function and autophagic degradation, promoting lipid storage disorders (Svegliati-Baroni et al., 2019; Sinha, 2023; Gęgotek and Skrzydlewska, 2024).

Therefore, the search for antioxidant compounds capable of mitigating the effects of lipid peroxidation in the liver and protecting fatty acids has become a promising area of research. Particular emphasis is placed on plant-derived substances that have the ability to modulate metabolic pathways and reduce inflammation in hepatocytes (Huang et al., 2022; Olędzka and Czerwińska, 2023; Ksepka et al., 2025).

Bioactive natural products are popularly used for disease prevention or therapy in the form of functional foods, nutraceuticals, dietary supplements, or drug discovery efforts aiming at the development of new pharmaceuticals (Matin et al., 2024; Tzvetkov et al., 2024; Zhu et al., 2024). Astaxanthin, a xanthophyll carotenoid derived from *Haematococcus pluvialis*, has demonstrated superior antioxidant capacity compared to other carotenoids and traditional antioxidants like vitamin E. Its unique molecular structure enables it to quench singlet oxygen, inhibit lipid peroxidation, and protect membrane integrity by embedding across the phospholipid bilayer (Sayuti et al., 2023; Zhang et al., 2025). Similarly, kokum (*Garcinia indica*) is rich in bioactive compounds such as garcinol and hydroxycitric acid (HCA) (Lim et al., 2021). Garcinol exhibits potent free radical scavenging activity and has been shown to inhibit lipid peroxidation by reducing reactive oxygen species (ROS) generation and stabilizing mitochondrial function (Thoyajakshi et al., 2024). By stabilizing cellular redox homeostasis, reducing inflammatory signaling, and modulating lipid metabolism, these supplements may serve as promising candidates in the early intervention or adjunctive management of NAFLD and related hepatic pathologies (Ambati et al., 2014; Murillo et al., 2016; Lim et al., 2021; Ksepka et al., 2026).

Since NAFLD progression involves several interrelated pathological processes, including oxidative stress, lipid peroxidation, altered fatty acid composition, and impaired lysosomal lipid degradation, the combination of astaxanthin and kokum may provide a broader multi-target hepatoprotective effect than either compound alone. Against this background, this study was designed to investigate the effects of astaxanthin and kokum supplementation on lipid peroxidation levels, fatty acid concentrations, and lysosomal degradation processes in the liver of mice with diet-induced NAFLD.

## 2 Materials and methods

The experiment was conducted on 120 male outbred Swiss Webster mice, which were randomly assigned into two main groups: a control group (C; n = 60) receiving a standard diet and an experimental group subjected to a modified methionine- and choline-deficient (MCD) diet (Altromin Spezialfutter GmbH and Co. KG, Lage, Germany) (D; n = 60).

The animals were maintained at the Animal Facility of the Institute of Genetics and Animal Biotechnology, Polish Academy of Sciences (Jastrzębiec, Poland), under standard laboratory conditions (22 °C ± 1 °C; 12:12 h light/dark cycle) in conventional cages with *ad libitum* access to food and water. All experimental procedures were approved by the Local Ethical Committee for Animal Experiments (approval no. WAW2/092/2024, 24 July 2024, II LEC in Warsaw) and conducted in accordance with the institutional guidelines, the principles of laboratory animal care, and Directive 2010/63/EU on the protection of animals used for scientific purposes (Directive, 2010/63/eu of the european parliament and of the council of 22 September 2010 on the protection of animals used for scientific purposes Text with EEA relevance, 2010). The experiment was also performed in compliance with the ARRIVE guidelines (du Sert et al., 2020).

**FIGURE 1:**
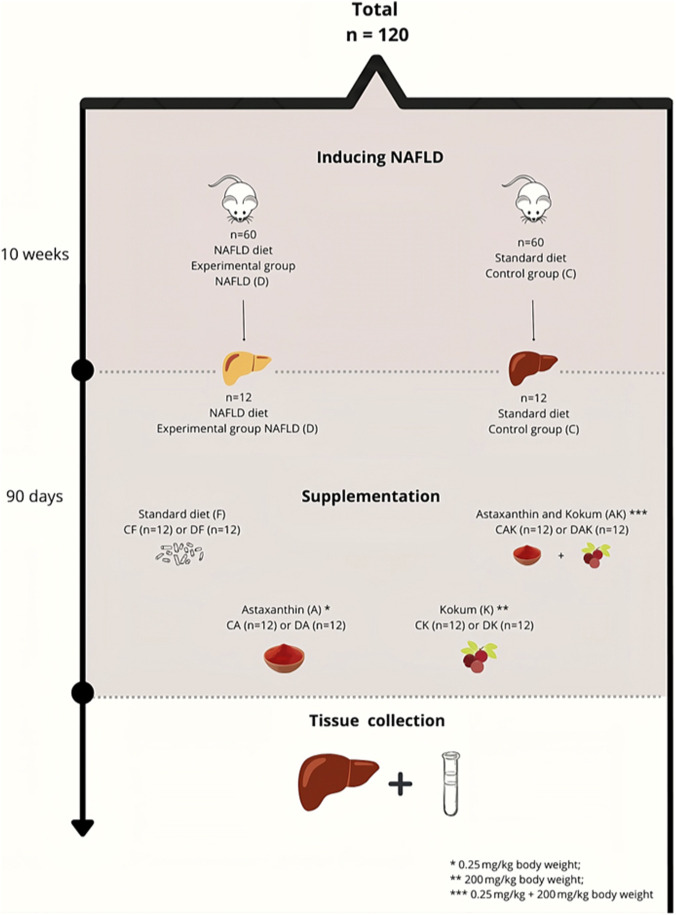
Experimental scheme of supplementation.

Mice from group D developed diet-induced NAFLD over 10 weeks. After this period, their diet was switched to a standard one. All animals from both C and D groups were then divided into subgroups (n = 12 per subgroup), according to the supplementation scheme (Figure 1). During the 90-day supplementation period, the continued control group without supplementation (CF) and the post-steatosis group without supplementation (DF) received a standard diet. The remaining subgroups received the same diet supplemented with astaxanthin at a dose of 0.25 mg/kg body weight (CA: non-steatotic group with astaxanthin supplementation; DA: steatotic group with astaxanthin supplementation), kokum at a dose of 200 mg/kg body weight (CK: non-steatotic group with kokum supplementation; DK: steatotic group with kokum supplementation), or their combination (CAK: non-steatotic group with combined supplementation; DAK: steatotic group with combined supplementation). The doses of astaxanthin and kokum were selected based on available literature data, safety considerations, and their biological and therapeutic relevance. Kokum was provided as *Garcinia indica* (Thouars) Choisy [Clusiaceae] fruit rind extract (Livinol™, Sabinsa Corporation, East Windsor, NJ, United States) standardized to contain 20% garcinol. The chemical profile of the extract was previously characterized by HPLC analysis (Majeed et al., 2020). Astaxanthin was used in the form of a water-dispersible microencapsulated powder containing 2.5% active compound derived from *Haematococcus pluvialis* (Algamo s. r.o., Mostek, Czech Republic). All supplemented feed was freshly prepared twice per week by thoroughly mixing the specified amounts of astaxanthin and/or kokum powder with standard laboratory chow before pellet formation. Fresh preparation and short-term storage in sealed containers prior to administration were applied to minimize potential degradation of the supplemented bioactive compounds.

At the end of the supplementation period, all animals were euthanized by decapitation (Leary and Johnson, 2020) in accordance with the approved ethical protocol. The procedure was carried out using a Rodent Guillotine type DCAP (Thermo Fisher Scientific, Waltham, MA, United States). Immediately after decapitation, liver tissues were excised and processed for subsequent analyses as described below.

### 2.1 Analytical procedure

#### 2.1.1 Determination of fatty acids

##### 2.1.1.1 Lipid extraction

Total lipids were extracted from mouse liver tissue using the Folch method with minor modifications (Folch et al., 1957). Briefly, approximately 100 mg of liver tissue was homogenized in 2 mL of ice-cold hexane/methanol (2:1, v/v) using a glass tissue grinder. After thorough homogenization, 0.5 mL of 0.88% KCl (w/v) solution was added to induce phase separation. The mixture was vortexed and centrifuged at 3,000 × g for 10 min at 4 °C. The lower (organic) phase was carefully collected and transferred to a clean glass vial. This step was repeated once with an additional 1 mL of hexane/methanol mixture to maximize lipid recovery. The pooled organic phases were evaporated to dryness under a gentle stream of nitrogen and stored at −80 °C until derivatization.

##### 2.1.1.2 Fatty acid methyl esters (FAME) derivatization and GC-MS/MS analysis

Dried lipid extracts were resuspended in 1 mL of hexane, followed by the addition of 200 µL of 2% (v/v) sulfuric acid in methanol. Samples were incubated at 80 °C for 90 min in sealed glass tubes to induce transmethylation. After cooling to room temperature, 1 mL of 0.9% NaCl and 1 mL of n-hexane were added to extract the FAMEs. The upper organic phase was collected, dried under nitrogen, and reconstituted in 100 µL of n-hexane containing methyl salicylate as an internal standard.

FAME standards (C6:0 to C24:1) and internal standard (methyl salicylate) were obtained from Sigma-Aldrich (St. Louis, MO, United States), with a purity >98%. All solvents were used of LC/MS grade. Methanol and n-hexane (LC/MS-grade) were purchased from Thermo Fisher Scientific (Swedesboro, NJ, United States). Other reagents were obtained from Sigma-Aldrich (St. Louis, MO, United States).

FAMEs were analyzed by gas chromatography with mass spectrometry (GC-MS, 7890A GC System, 5975C VL MSD with Triple-Axis Detector, Agilent Technologies) under conditions optimized for fatty acid profiling. Quantification was performed using calibration curves constructed from known concentrations of FAME (Supelco 37 Component FAME Mix) standards and normalized to the internal standard. Fatty acid concentrations were expressed as µg/mL of sample.

#### 2.1.2 Determination of malondialdehyde in liver tissue

Liver perfusion was performed with phosphate-buffered saline (PBS, pH 7.4) to remove residual blood. The perfused tissue was then homogenized in 2 mL of ice-cold phosphate buffer (pH 7.4), supplemented with 20 µL of butylated hydroxytoluene (BHT) in acetonitrile to inhibit *ex vivo* lipid peroxidation. Homogenization was carried out using a glass-Teflon homogenizer on ice. The homogenates were centrifuged at 10,000 × g for 10 min at 4 °C. The resulting supernatants were immediately placed on ice and used for the quantification of malondialdehyde.

MDA levels were measured using the OxisResearch™ Bioxytech® MDA-586™ assay kit, in accordance with the manufacturer’s protocol. The assay is based on the formation of a chromogenic adduct between N-methyl-2-phenylindole (NMPI) and MDA under acidic conditions at 45 °C. The reaction yields a stable, colored complex, which is specific for MDA and exhibits minimal cross-reactivity with other lipid peroxidation products.

Absorbance was measured at 586 nm using a Cary Varian 50 Bio UV-Vis spectrophotometer. MDA concentrations were determined using a standard curve constructed with known MDA concentrations and expressed as micromoles per liter (µM) of homogenate supernatant.

#### 2.1.3 Determination of lysosomal acid lipase (LAL) and lysosomal esterase (EL)

##### 2.1.3.1 Tissue preparation and homogenization

Mouse liver samples were weighed and immediately placed on ice. Tissues (∼100 mg) were homogenized in 1 mL of ice-cold homogenization buffer consisting of 50 mM sodium acetate (pH 4.5), 0.25 M sucrose, and 1 mM EDTA using a glass-Teflon Potter-Elvehjem homogenizer. The homogenates were centrifuged at 1,000 × g for 10 min at 4 °C to remove cell debris, and the supernatants were collected for enzymatic activity assays. Protein concentration was determined by the Bradford method (Bradford, 1976) using bovine serum albumin (BSA) as a standard.

##### 2.1.3.2 Lysosomal acid lipase activity assay

LAL activity was determined spectrophotometrically using phenolphthalein dibutyrate (PDP) as a chromogenic substrate. The enzymatic reaction was performed in 96-well microplates as follows:

- 50 µL of liver homogenate was added to 100 µL of substrate solution (1 mM PDP in 50 mM sodium acetate buffer, pH 4.5, containing 1% Triton X-100% and 1% isopropanol);
- The reaction mixture was incubated at 37 °C for 60 min.

After incubation, the reaction was stopped by adding 100 µL of stop solution (0.1 M sodium carbonate buffer, pH 10.4), which simultaneously allowed for color development of liberated phenolphthalein. The absorbance was measured at 550 nm using a plate reader (e.g., BioTek Synergy H1).

A standard curve was generated using known concentrations of free phenolphthalein to calculate the amount released. Enzyme activity was expressed as nmol phenolphthalein released per minute per milligram of protein (nmol/min/mg protein).

##### 2.1.3.3 Lysosomal esterase activity assay

Total EL activity was measured spectrophotometrically using p-nitrophenyl acetate (p-NPA) as a substrate. In brief, 20 µL of liver homogenate was added to 180 µL of assay buffer containing 50 mM Tris-HCl (pH 7.5), 0.5 mM p-NPA (Sigma-Aldrich), and 0.5% Triton X-100 in a 96-well clear flat-bottom plate. The reaction was monitored at 405 nm for 10–30 min at 25 °C using a plate reader. The change in absorbance was recorded kinetically. Results were normalized to protein content and expressed as nmol/min/mg protein.

### 2.2 Statistical analysis

Prior to performing one-way ANOVA, normality of the data distribution was assessed using D’Agostino-Pearson test, followed by validation of variances homogeneity with the Brown-Forsythe test. One-way ANOVA was applied to assess differences in fatty acid composition and lipid indices between experimental groups. Tukey’s *post hoc* test was used to identify specific group differences when ANOVA results were statistically significant (p < 0.05).

Pearson correlation analysis was performed to evaluate linear relationships between biochemical parameters, with correlation strength classified as weak (|r| < 0.3), moderate (0.3 ≤ |r| < 0.7), or strong (|r| ≥ 0.7).

## 3 Results

### 3.1 Fatty acid profile

The analysis of hepatic fatty acid profiles was conducted to compare the content of individual fatty acid fractions between control groups and groups with diet-induced NAFLD, with particular attention to the effects of astaxanthin and kokum supplementation administered separately or in combination.

In non-steatotic groups maintained on a standard diet, the initial control (C) and continued control (CF) groups served as baselines for assessing the potential influence of dietary supplementation. In the CF, CA, CK, and CAK groups, variations in the concentrations of selected saturated fatty acids (SFA) and unsaturated fatty acids (UFA) were observed.

In contrast, the steatotic group (D) exhibited pronounced alterations in hepatic fatty acid composition compared with the control group. Dietary normalization (DF) as well as supplementation with astaxanthin (DA), kokum (DK), or their combination (DAK) was associated with modifications of the hepatic fatty acid profile relative to group D, including lower levels of selected SFA and higher levels of monounsaturated (MUFA) and polyunsaturated fatty acids (PUFA).

Statistically significant differences between experimental groups (p < 0.05) were observed for the majority of the analyzed fatty acids (Table 1). All saturated and polyunsaturated fatty acids demonstrated statistically significant variation between groups. Among the monounsaturated fatty acids, significant differences were not observed only for C17:1 and C22:1n9. A detailed quantitative analysis of individual fatty acid species across experimental groups is presented in Table 1.

**TABLE 1:** Concentration of fatty acids (µg/mL) in mice liver.

Fatty acid	​	C	D	CF	DF	CA	DA	CK	DK	CAK	DAK	F-value	p-value
C12:0	LSMean	0.41^B^	122.02^A^	0.34^B^	1.11^C^	1.81^C^	1.72^C^	0.57^B^	1.02^C^	0.55^B^	1.21^C^	66.67	0.001
	± SD	0.14	39.12	0.12	0.66	0.84	0.75	0.17	0.59	0.21	0.74		
C14:0	LSMean	30.19^B^	431.84^A^	18.95^C^	27.26^B^	55.45^D^	48.27^D^	35.27^B^	36.63^B^	26.92^B^	63.62^D^	39.12	0.001
	± SD	11.48	119.65	8.75	9.78	21.30	14.98	9.48	7.41	8.97	22.12		
C14:1	LSMean	0.91^B^	5.68^A^	1.04^B^	0.22^C^	2.43^D^	1.02^B^	0.32^C^	2.18^D^	1.29^B^	2.99^D^	3.23	0.003
	± SD	0.35	2.10	0.39	0.70	1.11	0.72	0.11	1.63	0.79	1.12		
C15:0	LSMean	3.62^B^	8.62^A^	4.71^B^	5.24^AB^	8.67^A^	7.83^A^	7.52^A^	7.81^A^	6.27^AB^	8.58^A^	3.92	0.001
	± SD	0.82	3.35	1.27	1.39	2.93	1.13	1.40	2.87	1.72	2.12		
C16:0	LSMean	1676.35^B^	3958.22^A^	1529.22^B^	1756.80^B^	2413.46^AB^	2928.15^AB^	2461.56^AB^	2298.12^AB^	1709.08^AB^	3201.38^AB^	4.74	0.001
	± SD	331.68	1394.26	513.99	314.63	555.07	837.98	415.97	435.53	377.45	764.35		
C16:1	LSMean	353.47^B^	27.63^A^	295.57^B^	318.62^B^	538.16^C^	472.64^C^	374.05^B^	371.03^B^	317.57^B^	592.46^C^	3.11	0.004
	± SD	57.88	8.98	70.27	62.97	96.94	77.26	80.47	54.02	71.90	103.01		
C17:0	LSMean	8.83^C^	20.27^A^	12.13^B^	14.49^B^	18.75^A^	20.28^A^	19.81^A^	22.51^A^	15.26^B^	20.63^A^	6.37	0.001
	± SD	2.34	8.57	3.55	1.88	2.91	2.06	3.07	5.14	1.96	7.26		
C17:1	LSMean	1.33^B^	0.29^A^	0.22^A^	0.27^A^	2.82^C^	4.70^D^	2.78^C^	4.03^D^	0.39^A^	9.85^E^	1.05	0.417
	± SD	0.70	0.07	0.08	0.07	1.08	1.18	1.07	1.33	0.06	3.82		
C18:0	LSMean	796.83^B^	1668.06^A^	867.93^B^	1090.85^CB^	1193.70^C^	1403.17^A^	1380.25^A^	1528.96^A^	1025.90^BC^	1307.64^A^	9.54	0.001
	± SD	70.97	462.75	106.01	93.32	148.78	195.50	153.63	293.05	138.88	358.24		
C18:1cis (n9)	LSMean	243.44^B^	2165.61^A^	315.22^B^	305.14^B^	415.57^B^	1776.23^C^	464.26^B^	425.23^B^	346.98^B^	3998.17^D^	2.15	0.039
	± SD	75.64	647.58	88.62	93.93	87.50	773.18	119.57	149.15	105.63	793.23		
C18:2cis (n6)	LSMean	1080.26^B^	2446.90^A^	956.73^B^	1093.31^B^	1488.56^B^	1797.64^AB^	1509.56^B^	1407.43^B^	1050.22^B^	1947.00^AB^	4.80	0.001
	± SD	206.78	737.00	314.85	192.62	416.48	503.26	372.96	326.71	227.61	797.86		
C18:2	LSMean	0.06^B^	0.36^A^	0.08^B^	0.10^CB^	0.15^C^	0.26^D^	0.16^C^	0.14^C^	0.10^BC^	0.21^C^	2.53	0.016
	± SD	0.01	0.07	0.03	0.07	0.09	0.09	0.09	0.08	0.09	0.08		
C18:2trans (n6)	LSMean	1518.00^A^	1079.25^A^	1627.48^A^	1731.57^A^	2525.36^B^	1994.70^AB^	2118.31^AB^	2283.93^AB^	1999.73^AB^	2035.69^AB^	4.88	0.001
	± SD	218.93	217.84	642.78	314.76	726.79	117.45	313.10	604.02	485.07	530.82		
C20:0	LSMean	11.78^B^	30.01^A^	12.65^B^	19.71^C^	27.76^A^	27.67^A^	35.08^A^	28.26^A^	14.90^B^	28.89^A^	6.79	0.001
	± SD	2.13	9.74	3.09	1.26	7.24	4.24	9.21	7.25	2.91	9.20		
C18:3n6	LSMean	0.34^B^	1.19^A^	10.50^C^	7.21^C^	23.42^D^	13.33^C^	11.24^C^	13.66^C^	7.60^C^	4.86^D^	3.17	0.003
	± SD	0.16	0.54	2.05	2.43	9.44	3.86	4.08	4.73	2.68	1.06		
C20:1 (n9)	LSMean	20.62^A^	29.40^A^	30.28^A^	28.25^A^	63.25^B^	43.19^B^	50.74^B^	48.04^B^	36.79^A^	58.16^B^	2.49	0.018
	± SD	7.11	10.68	9.38	11.16	28.90	13.50	20.81	20.09	19.31	18.31		
C18:3n3	LSMean	8.05^B^	28.18^A^	27.91^A^	20.39^C^	59.14^D^	48.97^D^	54.09^D^	43.98^D^	23.15^A^	116.39^E^	2.42	0.020
	± SD	3.42	8.06	11.92	9.99	16.78	14.31	11.22	19.48	7.90	35.70		
C20:2	LSMean	1.42^B^	0.46^A^	9.02^C^	7.85^C^	11.57^C^	15.75^C^	19.89^D^	25.16^D^	11.48^C^	21.42^D^	8.78	0.001
	± SD	0.81	0.12	1.53	1.61	1.42	3.80	6.16	7.26	3.55	5.14		
C22:0	LSMean	0.10^B^	0.05^A^	0.05^A^	0.03^A^	0.09^B^	0.05^A^	0.06^A^	0.15^C^	0.03^A^	0.04^A^	2.75	0.009
	± SD	0.06	0.02	0.02	0.01	0.02	0.02	0.01	0.06	0.01	0.02		
C20:3n6	LSMean	33.66^B^	116.34^A^	71.19^C^	83.55^C^	127.52^A^	165.94^A^	156.32^A^	170.44^A^	105.22^A^	135.10^A^	19.63	0.001
	± SD	9.31	29.06	29.33	28.59	27.84	19.37	23.99	36.12	19.78	31.32		
C22:1n9	LSMean	0.54^A^	0.68^A^	0.64^A^	0.78^A^	0.78^A^	0.62^A^	1.02^A^	0.67^A^	0.74^A^	0.85^A^	0.68	0.723
	± SD	0.06	0.22	0.21	0.22	0.34	0.27	0.42	0.13	0.27	0.28		
C20:3n3	LSMean	551.71^A^	790.81^A^	772.94^A^	981.48^B^	1065.77^B^	1179.17^B^	1189.23^B^	1381.66^B^	939.43^B^	1040.34^B^	19.51	0.001
	± SD	80.97	95.98	220.94	101.74	118.96	42.63	134.08	268.28	110.20	117.27		
C20:4n6	LSMean	467.56^B^	704.01^A^	692.10^A^	888.45^A^	979.29^A^	1096.83^A^	1118.78^C^	1304.62^C^	879.77^A^	984.54^A^	21.07	0.001
	± SD	60.57	94.42	210.05	101.00	115.40	43.11	131.04	264.98	112.34	118.95		
C24:1	LSMean	0.08^A^	0.08^A^	0.17^A^	0.23^B^	0.28^B^	0.34^B^	0.38^B^	0.32^B^	0.41^B^	0.37^B^	4.35	0.001
	± SD	0.01	0.01	0.09	0.11	0.11	0.12	0.08	0.14	0.16	0.13		

Fatty acid abbreviations: C12:0 - lauric acid; C14:0 - myristic acid; C14:1 - myristoleic acid; C15:0 - pentadecanoic acid; C16:0 - palmitic acid; C16:1 - palmitoleic acid; C17:0 - margaric acid; C17:1 - heptadecenoic acid; C18:0 - stearic acid; C18:1cis (n9) - oleic acid; C18:2cis (n6) - linoleic acid; C18:2 - conjugated linoleic acid (CLA); C18:2trans (n6) - linolelaidic acid; C20:0 - arachidic acid; C18:3n6 - γ-linolenic acid; C20:1 (n9) - eicosenoic acid; C18:3n3 - α-linolenic acid; C20:2 - eicosadienoic acid; C22:0 - behenic acid; C20:3n6 - dihomo-γ-linolenic acid; C22:1n9 - erucic acid; C20:3n3 - eicosatrienoic acid; C20:4n6 - arachidonic acid; C24:1 - nervonic acid.

LSMean, Least Squares Mean; SD, Standard Deviation; Groups marked with different letters (e.g., A vs. B) differ significantly (p < 0.05); C - initial control group; D - hepatic steatosis group; CF, continued control group; DF, post-steatosis group; CA, control group supplemented with astaxanthin; DA, hepatic steatosis group supplemented with astaxanthin; CK, control group supplemented with kokum; DK, hepatic steatosis group supplemented with kokum; CAK, control group with combined supplementation; DAK, hepatic steatosis group with combined supplementation.

In addition to the analysis of individual fatty acid species, the total content of SFA and UFA was summarized. UFA were further subdivided into MUFA and PUFA. Ratios between SFA and UFA, MUFA, and PUFA, were calculated to assess the balance between saturated and unsaturated fatty acid pools. The PUFA/MUFA ratio was also determined as an indicator of the degree of unsaturation shift within the UFA fraction. The results are summarized in Table 2.

**TABLE 2:** Concentration of saturated, unsaturated fatty acids (µg/mL) and ratio of FA in mice liver.

Fatty acid fraction/ratio		C	D	CF	DF	CA	DA	CK	DK	CAK	DAK	F-value	p-value
SFA	LSMean	2528.10^B^	6239.09^A^	2445.98^B^	2915.47^B^	3719.68^C^	4437.12^C^	3940.12^C^	3923.44^C^	2798.90^B^	4631.99^C^	6.35	0.001
	± SD	364.59	1054.90	522.48	397.80	860.92	1031.65	635.67	627.13	424.06	599.17		
UFA	LSMean	4281.45^B^	7396.88^A^	4811.10^B^	5467.40^B^	7304.04^A^	8611.33^A^	7071.13^A^	7482.53^A^	5720.88^B^	10948.39^C^	1.80	0.087
	± SD	634.58	809.28	1136.15	682.54	1118.56	1615.14	1200.85	722.02	1062.12	2532.14		
MUFA	LSMean	620.40^B^	2229.38^A^	643.14^B^	653.50^B^	1023.27^C^	2298.73^A^	893.56^D^	851.51^D^	704.17^BD^	4662.83^E^	1.55	0.150
	± SD	271.25	465.72	179.19	295.78	299.75	462.73	223.27	238.32	303.20	660.14		
PUFA	LSMean	3661.05^B^	5167.51^A^	4167.96^C^	4813.90^AC^	6280.78^D^	6312.59^D^	6177.57^AD^	6631,02^D^	5016,71^A^	6285,56^D^	4.74	0.001
	± SD	441.76	766.50	716.30	657.66	836.21	620.77	838.66	802.39	967.02	801.98		
UFA/SFA	LSMean	1.69^B^	1.19^A^	1.97^B^	1.88^B^	1.96^B^	1.94^B^	1.79^B^	1,91^B^	2,04^B^	2,36^B^	16.01	0.001
	± SD	0.09	0.09	0.10	0.05	0.09	0.14	0.04	0.07	0.09	0.35		
MUFA/SFA	LSMean	0.25^B^	0.36^A^	0.26^B^	0.22^B^	0.28^B^	0.52^C^	0.23^B^	0.22^B^	0.25^B^	1.01^D^	2.57	0.014
	± SD	0.09	0.03	0.06	0.08	0.04	0.05	0.06	0.08	0.08	0.06		
PUFA/SFA	LSMean	1.45^B^	0.83^A^	1.70^B^	1.65^B^	1.69^B^	1.42^B^	1.57^B^	1.69^B^	1.79^B^	1.36^B^	36.59	0.001
	± SD	0.08	0.06	0.13	0.07	0.06	0.13	0.10	0.05	0.05	0.16		
PUFA/MUFA	LSMean	5.90^B^	2.32^A^	6.48^B^	7.37^B^	6.14^B^	2.75^A^	6.91^B^	7.79^B^	7.12^B^	1.35^C^	4.18	0.001
	± SD	0.63	0.55	0.87	0.89	0.75	1.26	1.05	0.45	0.99	0.75		

LSMean, Least Squares Mean; SD, Standard Deviation; Groups marked with different letters (e.g., A vs. B) differ significantly (p < 0.05); C - initial control group; D - hepatic steatosis group; CF, continued control group; DF, post-steatosis group; CA, control group supplemented with astaxanthin; DA, hepatic steatosis group supplemented with astaxanthin; CK, control group supplemented with kokum; DK, hepatic steatosis group supplemented with kokum; CAK, control group with combined supplementation; DAK, hepatic steatosis group with combined supplementation.

The highest overall content of SFA was observed in steatotic group (D). Compared with this group, SFA levels decreased in all post-intervention groups. The highest total UFA content was observed in the DAK group. The UFA/SFA and PUFA/SFA ratios decreased in group D compared with group C. The greatest increase in the UFA/SFA and MUFA/SFA ratios was observed in steatotic group with combined supplementation (DAK), whereas the highest PUFA/SFA ratio among steatotic groups was found in the DF and DK group (Table 2).

**FIGURE 2:**
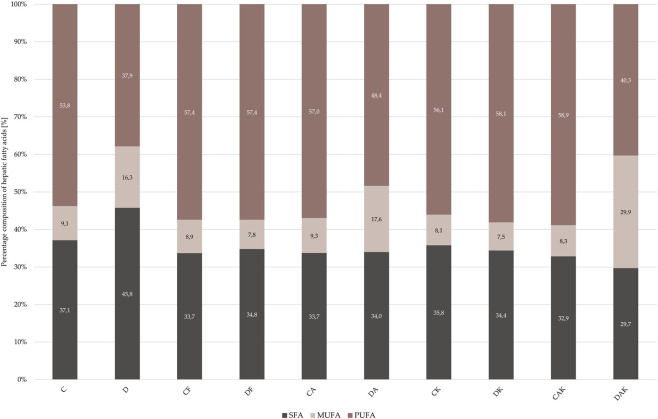
Percentage profile of fatty acids SFA, MUFA and PUFA in all experimental groups; C - initial control group; D - hepatic steatosis group; CF - continued control group; DF - post-steatosis group; CA - control group supplemented with astaxanthin; DA - hepatic steatosis group supplemented with astaxanthin; CK - control group supplemented with kokum; DK - hepatic steatosis group supplemented with kokum; CAK - control group with combined supplementation; DAK - hepatic steatosis group with combined supplementation.

Figure 2 presents changes in the percentage composition of hepatic fatty acid classes, including SFA, MUFA, and PUFA across all experimental groups. The data reveal marked group-dependent differences in fatty acid distribution.

Group D exhibited the highest proportion of SFA (45.8%) and the lowest UFA content (54.2%), indicating a shift toward a more saturated hepatic lipid profile, typically associated with steatotic and pro-oxidative conditions. In contrast, all supplemented groups showed a reduction in SFA, with the lowest level observed in the DAK group (29.7%). Furthermore, in this group, the balance among fatty acid types favored MUFA and PUFA, suggesting a shift in the liver fatty acid composition toward more unsaturated and potentially antioxidant profiles. This may reflect a synergistic effect of astaxanthin and kokum in restoring a more favorable fatty acid balance under metabolically compromised conditions.

### 3.2 Degradation and oxidation of lipids fraction

The activities of lysosomal esterase and lysosomal acid lipase, as well as malondialdehyde concentration, as an index of lipid peroxidation, were evaluated in liver homogenates across all experimental groups. Statistically significant differences were observed for all three parameters (p < 0.001; Tables 3, 4).

**TABLE 3:** Activity (nmol/mg protein per h) of lysosomal enzymes (esterase and lipase).

​	EL		LAL	
Group	LSMean	± SD	LSMean	± SD
C	357.35^B^	34.61	47.05^B^	3.10
D	553.86^A^	43.32	68.62^A^	6.31
CF	340.63^B^	21.30	45.96^B^	2.34
DF	365.42^B^	28.80	46.59^B^	3.59
CA	290.97^B^	31.33	47.16^B^	4.89
DA	379.82^B^	31.04	65.39^A^	5.36
CK	289.60^B^	21.27	44.92^B^	2.69
DK	351.02^B^	19.39	59.64^A^	1.38
CAK	303.45^B^	47.45	45.57^B^	5.06
DAK	362.31^B^	32.38	54.50^A^	6.73
F-value	38.32	​	28.03	​
p-value	0.001	​	0.001	​

LSMean, Least Squares Mean; SD, Standard Deviation; Groups marked with different letters (e.g., A vs. B) differ significantly (p < 0.05); C - initial control group; D - hepatic steatosis group; CF, continued control group; DF, post-steatosis group; CA, control group supplemented with astaxanthin; DA, hepatic steatosis group supplemented with astaxanthin; CK, control group supplemented with kokum; DK, hepatic steatosis group supplemented with kokum; CAK, control group with combined supplementation; DAK, hepatic steatosis group with combined supplementation.

**TABLE 4:** Concentration (µmol/L) of malondialdehyde, lipid peroxidation marker.

​	MDA	
Group	LSMean	± SD
C	34.89^B^	4.5
D	49.67^A^	8.29
CF	32.15^B^	0.97
DF	36.55^B^	2.55
CA	30.81^B^	3.57
DA	33.77^B^	4.21
CK	25.47^B^	4.52
DK	27.26^B^	2.63
CAK	18.40^C^	3.51
DAK	25.45^B^	2.97
F-value	28.17	​
p-value	0.001	​

LSMean, Least Squares Mean; SD, Standard Deviation; Groups marked with different letters (e.g., A vs. B) differ significantly (p < 0.05); C - initial control group; D - hepatic steatosis group; CF, continued control group; DF, post-steatosis group; CA, control group supplemented with astaxanthin; DA, hepatic steatosis group supplemented with astaxanthin; CK, control group supplemented with kokum; DK, hepatic steatosis group supplemented with kokum; CAK, control group with combined supplementation; DAK, hepatic steatosis group with combined supplementation.

The highest activities of both lysosomal enzymes were observed in group D (Table 3). In mice with steatosis, dietary normalization (DF) and supplementation with astaxanthin, kokum, or their combination were associated with lower esterase activity relative to group D, with the most pronounced normalization observed in the kokum-supplemented group. For LAL, dietary normalization (DF) restored lipase activity to the control range (C), whereas supplementation initiated after steatosis induction (DA, DK, DAK) was associated with higher lipase activity than in control groups.

The highest hepatic MDA levels were observed in the diet-induced NAFLD group (D), while the lowest values were recorded in the CAK group (Table 4). Among steatotic groups, dietary normalization (DF) and supplementation with astaxanthin, kokum, or their combination were associated with reduced MDA levels compared with group D, with the strongest effect observed in the DAK group.

**FIGURE 3:**
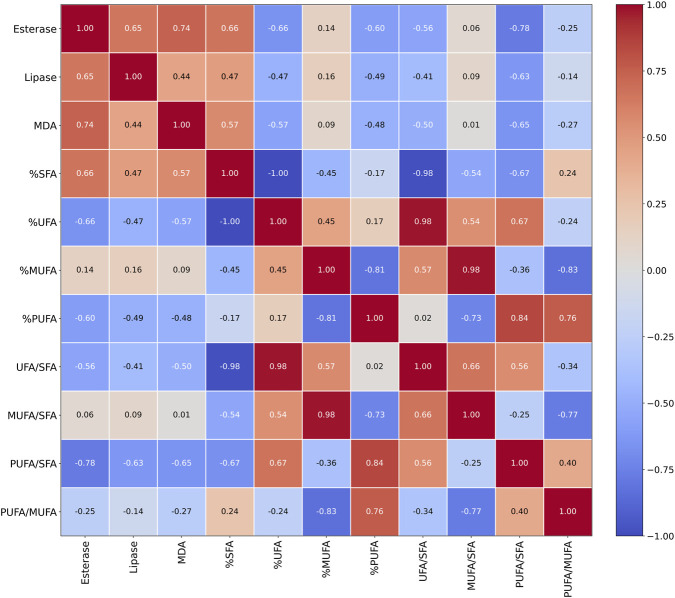
Correlation between all groups. Note: Red shading indicates a positive correlation, while blue denotes a negative correlation. The intensity of the color corresponds to the strength of the correlation, with darker hues representing stronger associations.

In addition, correlation analysis was performed to investigate the relationships between lysosomal enzyme activities (LAL and EL), MDA levels, and total contents of SFA, UFA, MUFA and PUFA, as well as their ratios, to further explore potential associations between lysosomal lipid degradation capacity and hepatic lipid composition and oxidative stress (Figure 3).

Correlation analysis indicated relationships between lysosomal enzymes activities (EL, LAL), lipid peroxidation (MDA), and fatty acid composition. A strong positive correlation was observed between esterase activity and MDA level (r = 0.74), and a moderate positive correlation with %SFA (r = 0.66). Negative correlations were found with %UFA (r = −0.66) and %PUFA (r = −0.60). The strongest inverse association for EL was observed with the PUFA/SFA ratio (r = −0.78). LAL activity was moderately positively correlated with MDA (r = 0.44) and %SFA (r = 0.47), and negatively correlated with %UFA (r = −0.47), %PUFA (r = −0.49), and PUFA/SFA (r = −0.63). MDA was positively correlated with %SFA (r = 0.57) and negatively with %UFA (r = −0.57), %PUFA (r = −0.48), UFA/SFA (r = −0.50), and PUFA/SFA (r = −0.65). A strong negative correlation was also found between %MUFA and %PUFA (r = −0.81). The PUFA/MUFA ratio was strongly positively correlated with %PUFA (r = 0.76) and negatively with MUFA/SFA (r = −0.77). Moreover, %UFA was very strongly positively correlated with the UFA/SFA ratio (r = 0.98), and %MUFA was likewise very strongly correlated with the MUFA/SFA ratio (r = 0.98).

## 4 Discussion

This study presents a comprehensive assessment of hepatic fatty acid profile, lipid peroxidation, and lysosomal enzyme activity in a murine model of diet-induced NAFLD, along with the therapeutic impact of antioxidant supplementation with astaxanthin and kokum. By integrating lipidomic, oxidative, and enzymatic parameters, the present work characterizes biochemical alterations associated with hepatic steatosis and their modulation by nutritional interventions.

Consistent with previous studies describing the “multiple-hit” model of NAFLD progression (Guo et al., 2022), mice fed a methionine- and choline-deficient diet (group D) developed a pathological hepatic lipid profile characterized by a significant increase in saturated fatty acids, accompanied by a reduction in the UFA/SFA ratio. These changes are in agreement with previous reports describing impaired fatty acid handling and enhanced lipotoxicity during steatosis (Alves-Bezerra and Cohen, 2018; Heeren and Scheja, 2021). Importantly, Xu et al. (2024) reported that although MUFAs are more likely to promote the accumulation of lipids and triglycerides in hepatocytes, SFAs are more strongly associated with hepatotoxic effects.

These lipid alterations were accompanied by elevated hepatic MDA levels in this group, indicating enhanced lipid peroxidation and oxidative stress, which are recognized contributors to NAFLD progression (Ayala et al., 2014; Powell et al., 2021). Lipid peroxidation, primarily driven by hydroxyl and hydroperoxyl radicals, initiates membrane destabilization and propagates oxidative damage through reactive aldehydes such as MDA and 4-hydroxy-2-nonenal (4-HNE). MDA is not only a marker but also a mediator of cellular dysfunction, capable of forming protein and DNA adducts that impair enzymatic function and promote fibrogenesis (Ayala et al., 2014).

In parallel with these changes, activities of lysosomal enzymes - EL and LAL - were increased in animals with liver steatosis. It may reflect intensified hepatocellular turnover and lysosomal activation, in response to lipid overload and oxidative stress (Bradić et al., 2023). Similar increases in lysosomal enzyme activity have been reported in experimental models of hepatic steatosis and have been interpreted as adaptive changes in intracellular lipid processing under conditions of excessive substrate availability (Strebinger et al., 2019; Zeng et al., 2023).

Lysosomal enzymes, particularly LAL, play a crucial role in hepatic lipid homeostasis through the hydrolysis of triglycerides and cholesteryl esters within lysosomes. In contrast, several studies indicate that reduced LAL activity accompanies progressive NAFLD and NASH, correlating with hepatic lipid accumulation, systemic inflammation, and dyslipidemia (Baratta et al., 2019; Carotti et al., 2020; Thoen et al., 2021). This suggests that the elevated LAL activity observed in group D may represent a compensatory lysosomal response occurring at an earlier stage of lipid overload rather than a feature of advanced disease. In contrast, prolonged disease progression in advanced NAFLD may eventually lead to lysosomal dysfunction and impaired lipid degradation efficiency.

In the present study, dietary normalization reduced both EL and LAL activities, with LAL reaching values comparable with those observed in the control group. This was accompanied by a significant decrease in liver MDA, indicating attenuation of oxidative stress. Importantly, the fatty acid profile improved in this group, as demonstrated by a significant decrease in SFA and an improvement in the UFA/SFA ratio compared to the steatosis group (D). This observation suggests that dietary normalization partially reverses steatosis-induced hepatic alterations; however, the magnitude of these changes appears less pronounced than in the supplemented groups.

In the steatotic groups, antioxidant supplementation led to attenuation of lipid peroxidation and modification of hepatic fatty acid composition. Compared with the non-supplemented steatotic group, all supplemented groups (DA, DK, and DAK) exhibited reduced MDA levels and lower lysosomal esterase activity, indicating alleviation of oxidative stress and decreased lysosomal involvement in lipid degradation processes. These changes were accompanied by a shift in hepatic fatty acid composition toward a higher proportion of unsaturated fatty acids, suggesting a transition toward a less lipotoxic hepatic lipid profile. Astaxanthin supplementation, which resulted in decreased MDA levels and esterase activity, is consistent with previous reports describing its role in reactive oxygen species scavenging and modulation of hepatic lipid metabolism (Guerra and Otton, 2011; Jia et al., 2016; Wu et al., 2020). Kokum exerted an equally pronounced, and in some parameters even stronger effect, supporting the antioxidant and anti-inflammatory properties of its bioactive components (Majeed et al., 2020). Notably, combined supplementation (DAK) was associated with the most pronounced reduction in lipid peroxidation together with a marked increase in UFA and MUFA content and improvement in the UFA/SFA and MUFA/SFA ratios. Despite the fact that the absolute SFA content in that group remained relatively high compared with the other supplemented groups, its relative contribution to the total hepatic fatty acid pool was the lowest among all groups, reflecting a marked increase in unsaturated fatty acids. This shift suggests a transition toward a less lipotoxic hepatic lipid profile. This observation is consistent with clinical evidence indicating that alterations in hepatic fatty acid profiles are associated with the severity of NAFLD and histopathological changes (Yamada et al., 2015). Furthermore, it has been demonstrated that fatty acid composition and the balance between saturated and unsaturated fractions, rather than their quantity, are pivotal factors determining hepatic steatosis (Das et al., 2023).

In healthy animals maintained on a standard diet, metabolic parameters remained stable. Moreover, no significant differences were observed in esterase or lipase activities among the supplemented control groups, indicating that under physiological conditions lysosomal lipid degradation remains relatively constant and is not substantially modulated by supplementation. In contrast, supplementation exerted a clear effect on lipid peroxidation. Among the control groups, the lowest MDA levels were recorded in the CAK group, suggesting a synergistic antioxidant effect of astaxanthin and kokum. This effect may be attributed to the complementary action of astaxanthin within the lipid environment and bioactive constituents of kokum, such as garcinol and anthocyanins, which together may attenuate lipid peroxidation and pro-inflammatory processes even under physiological conditions (Lim et al., 2021; Medoro et al., 2024).

The beneficial effect of combined supplementation may also be associated with the distinct yet interacting biological activities of astaxanthin and kokum phytochemicals. Astaxanthin may primarily protect lipid membranes against peroxidation and modulate antioxidant and metabolic pathways, including Nrf2/HO-1, NF-κB, and AMPK/SIRT1 signaling (Li et al., 2022). Kokum-derived garcinol and hydroxycitric acid may additionally suppress ROS generation, inhibit NF-κB/MAPK-mediated inflammation, and reduce *de novo* lipogenesis through inhibition of ATP citrate lyase (Chang et al., 2021). Therefore, the combination may act at several interconnected levels, including oxidative stress, inflammation, lipid peroxidation, and hepatic fatty acid metabolism. This interpretation is consistent with the lowest overall MDA levels observed in the CAK group, as well as the strongest reduction in lipid peroxidation among steatotic animals in the DAK group. Moreover, combined supplementation in both CAK and DAK groups was associated with a favorable shift toward a higher proportion of unsaturated fatty acids. Nevertheless, as these pathways were not directly evaluated in the present study, the proposed mechanism remains hypothetical and requires further investigation.

A limitation of the present study is that the MCD model, although effective in inducing hepatic steatosis and oxidative stress, does not fully reproduce the metabolic background of human NAFLD, particularly obesity, weight gain, and insulin resistance. Therefore, the present findings should be interpreted in light of the specific features of this experimental model.

## 5 Conclusion

Collectively, the findings of this study underscore the importance of integrating lipidomic, oxidative, and enzymatic markers for a multidimensional understanding of NAFLD progression and its modulation by dietary interventions. The accumulation of saturated fatty acids, elevation of malondialdehyde, and increased lysosomal enzyme activity form a pathophysiological triad characteristic of non-alcoholic fatty liver disease. Dietary normalization partially improved these alterations, whereas antioxidant supplementation further reduced oxidative stress and modified hepatic fatty acid profiles toward a higher proportion of unsaturated fatty acids. Importantly, combined supplementation with astaxanthin and kokum resulted in the strongest reduction in lipid peroxidation together with favorable changes in fatty acid composition, including increased UFA and MUFA content and improved UFA/SFA and MUFA/SFA ratios. These findings highlight the potential of astaxanthin and kokum as complementary nutraceuticals capable of modulating hepatic lipid composition and oxidative balance, thereby contributing to the mitigation of diet-induced steatosis.

## Data Availability

The original contributions presented in the study are included in the article/supplementary material, further inquiries can be directed to the corresponding authors.

## Glossary

MASLD: Metabolic dysfunction-associated steatotic liver disease
NAFLD: Non-alcoholic fatty liver disease
FAME: Fatty acid methyl esters
MUFA: Monounsaturated fatty acids
NASH: Non-alcoholic steatohepatitis
NMPI: N-methyl-2-phenylindole
PUFA: Polyunsaturated fatty acids
VLDL: Very low-density lipoproteins
BHT: Butylated hydroxytoluene
BSA: Bovine serum albumin
CAK: Non-steatotic group with combined supplementation
DAK: Steatotic group with combined supplementation
FFA: Free fatty acids
HCA: Hydroxycitric acid
LAL: Lysosomal acid lipase
MCD: Methionine- and choline-deficient
MDA: Malondialdehyde
NPA: Nitrophenyl acetate
PDP: Phenolphthalein dibutyrate
ROS: Reactive oxygen species
SFA: Saturated fatty acids
UFA: Unsaturated fatty acids
CA: Non-steatotic group with astaxanthin supplementation
CE: Cholesterol esters
CF: Continued control group without supplementation
CK: Non-steatotic group with kokum supplementation
DA: Steatotic group with astaxanthin supplementation
DK: Steatotic group with kokum supplementation
DF: Post-steatosis group without supplementation
EL: Lysosomal esterase
FA: Fatty acids
TG: Triglycerides
C: Initial control group
D: Steatotic group.
